# Classification of microarray data using gene networks

**DOI:** 10.1186/1471-2105-8-35

**Published:** 2007-02-01

**Authors:** Franck Rapaport, Andrei Zinovyev, Marie Dutreix, Emmanuel Barillot, Jean-Philippe Vert

**Affiliations:** 1 lnstitut Curie, Service de Bioinformatique, 26 rue d'Ulm, F-75248 Paris Cedex 05, France; 2 Ecole des Mines de Paris, Centre for Computational Biology, 35 rue Saint-Honoré, 77300 Fontainebleau, France; 3 lnstitut Curie, CNRS-UMR 2027, Bâtiment 110, Centre Universitaire, F-91405 Orsay, France

## Abstract

**Background:**

Microarrays have become extremely useful for analysing genetic phenomena, but establishing a relation between microarray analysis results (typically a list of genes) and their biological significance is often difficult. Currently, the standard approach is to map *a posteriori *the results onto gene networks in order to elucidate the functions perturbed at the level of pathways. However, integrating *a priori *knowledge of the gene networks could help in the statistical analysis of gene expression data and in their biological interpretation.

**Results:**

We propose a method to integrate *a priori *the knowledge of a gene network in the analysis of gene expression data. The approach is based on the spectral decomposition of gene expression profiles with respect to the eigenfunctions of the graph, resulting in an attenuation of the high-frequency components of the expression profiles with respect to the topology of the graph. We show how to derive unsupervised and supervised classification algorithms of expression profiles, resulting in classifiers with biological relevance. We illustrate the method with the analysis of a set of expression profiles from irradiated and non-irradiated yeast strains.

**Conclusion:**

Including *a priori *knowledge of a gene network for the analysis of gene expression data leads to good classification performance and improved interpretability of the results.

## Background

During the last decade microarrays have become the technology of choice for dissecting the genes responsible for a phenotype. By monitoring the activity of virtually all the genes from a sample in a single experiment they offer a unique perspective for explaining the global genetic picture of a variant, whether a diseased individual or a sample subject to whatever stressing conditions. However, this strength is also their major weakness, and has led to the "gene list" syndrome. Following careful experimental design and data analysis, the result of an experiment with microarrays is often summarized as a list of genes that are differentially expressed between two conditions, or that allow samples to be classified according to their phenotypic features. Once this list of genes, typically a few hundreds, as been obtained, its meaning still has to be deciphered, but the automated translation of the list into biological interpretation is often challenging.

The interpretation of the results in terms of biological functions and pathways involving several genes is of particular interest. Many databases and tools help verify *a posteriori *whether genes known to co-operate in some biological process are found in the list of genes selected. For example, Gene Ontology [1], Biocarta [2], GenMAPP [3] and KEGG [4] all allow a list of genes to be crossed with biological functions and genetic networks, including metabolic, signalling or other regulation pathways. Basic statistical analysis (e.g., [5,6]) can then determine whether a pathway is over-represented in the list, and whether it is over-activated or under-activated. However, one can argue that introducing information on the pathway at this point in the analysis process sacrifices some statistical power to the simplicity of the approach. For example, a small but coherent difference in the expression of all the genes in a pathway should be more significant than a larger difference occurring in unrelated genes.

There is therefore a pressing need for methods integrating *a priori *pathway knowledge in the gene expression analysis process, and several attempts have been carried out in that direction so far. Several authors have used *a priori *known gene networks to derive models and constraints for gene expression. For example, logical discrete formalism [7] can be used to analyse all the possible steady states of a biochemical reaction network described by positive and negative influences and can determine whether the observed gene expression may be explained by a perturbation of the network. If only the signs of the concentration differences between two steady states are considered, it is possible to solve the corresponding Laplace equation in sign algebra [8], giving qualitative predictions for the signs of the concentration differences measured by microarrays. Other approaches, such as the MetaReg formalism [9], have also been used to predict possible gene expression patterns from the network structure, although these approaches adhere less to the formal theory of biochemical reaction networks.

Unfortunately, methods based on network models are rarely satisfactory because detailed quantitative knowledge of the complete reaction network parameters is often lacking, or only fragments of the network structure are available. In these cases, more phenomenological approaches need to be used. Pathway scoring methods try to detect perturbated "modules" or network pathways while ignoring the detailed network topology (for recent reviews see [10,11]). It is assumed that the genes inside a module are co-ordinately expressed, and thus a perturbation is likely to affect many of them.

With available databases containing tens of thousands of reactions and interactions (KEGG [4], TransPath [12], BioCyc [13], Reactome [14] and others), the problem is how to integrate the detailed graph of gene interactions (and not just crude characteristics such as the inter/intra-module connectivity) into the core microarray data analysis. Some promising results have been reported with regard to this problem. [15] developed a method for correlating interaction graphs and different types of quantitative data, and [16] showed that explicitly taking the pathway distance between pairs of genes into account enhances the statistical scores when identifying activated pathways. The co-clustering of gene expression and gene networks has been reported [17], and a dimension reduction method, called "Network component analysis" [18,19], was proposed to construct linear models of gene regulation based on *a priori *known network information. The PATIKA project [20] proposed a score to quantify the compatibility of a pathway with a given microarray data, and in [21] a network topology extracted from literature was used jointly with microarray data to find significantly affected pathway regulators.

In this paper, we investigate a different approach for integrating gene network knowledge early in the gene expression analysis. By "gene network" we mean any graph with genes as vertices, and where edges between genes can represent various biological information. For example, an edge between two genes could represent the fact that their products interact physically (protein-protein interaction network), the presence of a genetic interaction such as a synthetic-lethal or suppressor interaction [22], or the fact that these genes code for enzymes that catalyse successive chemical reactions in a pathway (metabolic network, [15]). As an illustration we focus on the latter case in this article, although the method proposed is not limited to the metabolic network. Our approach is based on the biological hypothesis that genes close on the network are likely to have similar expression, and consequently that noisy measures of gene expression, such as those obtained by microarrays, can be denoised to some extent by extracting their "low-frequency" component on the gene network. In the case of the metabolic gene network of the yeast *S. cerevisiae *considered in this study, this biological hypothesis is motivated by previous observations that genes coding for enzymes involved in a common process are often co-regulated ensuring the presence of all the necessary proteins [15,17,23-25].

The approach is formally based on the spectral decomposition of the gene expression measurements with respect to the gene network seen as a graph, followed by an attenuation of the high-frequency components of the expression vectors with respect to the topology of the graph. We show how to derive unsupervised clustering and supervised classification algorithms for expression profiles, resulting in classifiers that can be easily interpreted in terms of pathways. We illustrate the relevance of our approach by analysing a gene expression dataset monitoring the transcriptional response of irradiated and non-irradiated yeast colonies [26]. We show that by filtering out 80% of the eigenmodes of the KEGG metabolic network in the gene expression profile, we obtain accurate and interpretable discriminative model that may lead to new biological insights.

### Data

We collected the expression data from a study analysing the effect of low irradiation doses on *Saccharomyces cerevisiae *strains [26]. The first group of extracted expression profiles was a set of twelve independent yeast cultures grown without radiation (not irradiated, NI). From this group, we excluded an outlier that the author of the article indicated to us. The second group was a set of six independent irradiated (I) cultures exposed to a dose of 15–20 mGy/h for 20 h. This dose of irradiation produces no mutagenic effects, but induces transcriptional changes. We used the same normalisation method as in the first study of this data (Splus LOWESS function, see [26] for details), then we attempted (1) to separate the NI samples from the I ones, and (2) to understand the difference between the two populations in terms of metabolic pathways.

The gene network model used to analyse the gene expression data was therefore built from the KEGG database of metabolic pathways [4]. The metabolic gene network is a graph in which the enzymes are vertices and the edges between two enzymes indicate that the product of a reaction catalysed by the first enzyme is the substrate of the reaction catalysed by the second enzyme. We reconstructed this network from the KGML v0.3 version of KEGG, resulting in 4694 edges between 737 genes. We kept only the largest connected component (containing 713 genes) for further spectral analysis.

## Results

### Unsupervised classification

**Figure 1:**
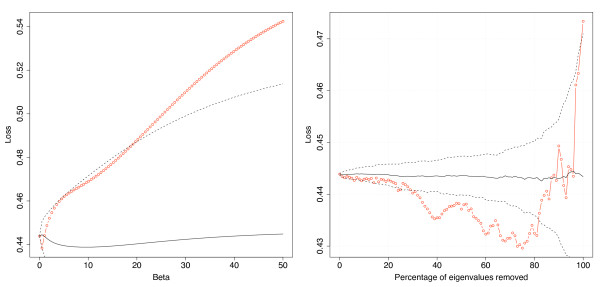
**Unsupervised classification results**. Performance of the unsupervised classification after changing the metric with the function *φ*(*λ*) = exp(-*βλ*) for different values of *β *(left), or with the function *φ*(*λ*) = 1(*λ *<*λ*_0_) with varying *λ*_0_, that is, by keeping a variable number of smallest eigenvalues (right). The red curve is obtained with the KEGG network. The black curves show the result (mean and one standard deviation interval) obtained with a random network.

First, we tested the general effect of modifying the distances between expression profiles using the KEGG metabolic pathways as background information in an unsupervised setting. We calculated the pairwise distances between all 17 expression profiles after applying the transformations defined by the filters (4) and (5), over a wide range of parameters. We assessed whether the resulting distances were more coherent with a biological intepretation by calculating the ratio of intraclass distances over all pairwise distances, defined by:

r=∑u1,v1∈V1d(u1,v1)2+∑u2,v2∈V2d(u2,v2)2∑u,v∈Vd(u,v)2,
 MathType@MTEF@5@5@+=feaafiart1ev1aaatCvAUfKttLearuWrP9MDH5MBPbIqV92AaeXatLxBI9gBaebbnrfifHhDYfgasaacH8akY=wiFfYdH8Gipec8Eeeu0xXdbba9frFj0=OqFfea0dXdd9vqai=hGuQ8kuc9pgc9s8qqaq=dirpe0xb9q8qiLsFr0=vr0=vr0dc8meaabaqaciaacaGaaeqabaqabeGadaaakeaacqWGYbGCcqGH9aqpdaWcaaqaamaaqababaGaemizaqMaeiikaGIaemyDau3aaSbaaSqaaiabigdaXaqabaGccqGGSaalcqWG2bGDdaWgaaWcbaGaeGymaedabeaakiabcMcaPmaaCaaaleqabaGaeGOmaidaaaqaaiabdwha1naaBaaameaacqaIXaqmaeqaaSGaeiilaWIaemODay3aaSbaaWqaaiabigdaXaqabaWccqGHiiIZcqWGwbGvdaWgaaadbaGaeGymaedabeaaaSqab0GaeyyeIuoakiabgUcaRmaaqababaGaemizaqMaeiikaGIaemyDau3aaSbaaSqaaiabikdaYaqabaGccqGGSaalcqWG2bGDdaWgaaWcbaGaeGOmaidabeaakiabcMcaPmaaCaaaleqabaGaeGOmaidaaaqaaiabdwha1naaBaaameaacqaIYaGmaeqaaSGaeiilaWIaemODay3aaSbaaWqaaiabikdaYaqabaWccqGHiiIZcqWGwbGvdaWgaaadbaGaeGOmaidabeaaaSqab0GaeyyeIuoaaOqaamaaqababaGaemizaqMaeiikaGIaemyDauNaeiilaWIaemODayNaeiykaKYaaWbaaSqabeaacqaIYaGmaaaabaGaemyDauNaeiilaWIaemODayNaeyicI4SaemOvayfabeqdcqGHris5aaaakiabcYcaSaaa@6D6A@

where *V*_1 _and *V*_2 _are the two classes of points. We compared the results with those obtained by replacing KEGG with a random network, produced by keeping the same graph structure but randomly permutating the vertices, in order to assess the significance of the results. We generated 100 such networks to give an average result with a standard deviation. Figure 1 shows the result for the function *φ*_exp_(*λ*) = exp(-*βλ*) with varying *β *(left), and for the function *φ*_thres_(*λ*) = 1(*λ *<*λ*_0_) for varying *λ*_0 _(right). We observe that, apart from very small values of *β*, the change of metric with the *φ*_exp _function performs worse than that of a random network. The second method (filtering out the high frequency components of the gene expression vector), in which up to 80% of the eigenvectors are removed, performs significantly better than that of a random network. When only the top 3% of the smoothest eigenvectors are kept, the performance is similar to that of a random network, and when only the top 1% is kept, the performance is significantly worse. This explains the disappointing results obtained with the *φ*_exp _function: by giving more weight to the small eigenvalues exponentially, the method focuses on those first few eigenvectors that, as shown by the second method, do not provide a geometry compatible with the separation of samples into two classes. From the second plot, we can infer that at least 20% of the KEGG eigenvectors should be given sufficient weight to obtain a geometry compatible with the classification of the data in this case.

### PCA analysis

We carried out a principal component analysis (PCA, [27]) on the original expression vectors *f *and compared this with a PCA of the transformed set of vectors *S*_*φ *_(*f*) obtained with the function *φ*_thres _to further investigate the effect of filtering out the high frequencies of the expression profiles on their relative positions.

**Figure 2:**
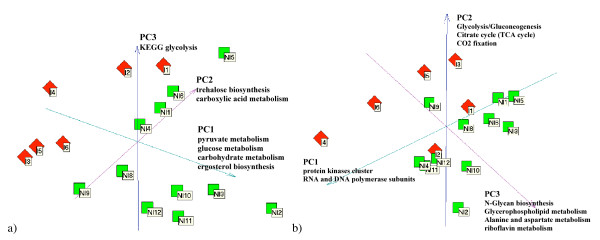
**PCA Plot**. PCA plots of the initial expression profiles (a) and the transformed profiles using network topology (80% of the eigenvalues removed) (b). The green squares are non-irradiated samples and the red rhombuses are irradiated samples. Individual sample labels are shown together with GO and KEGG annotations associated with each principal component.

Analysis of the initial sample distribution (Figure 2) shows that the first principal component can partially separate irradiated from non-irradiated samples, with exception of the two irradiated samples "I1" and "I2", as they have larger projections onto the third principal component than onto the first one. The experimental protocol revealed that these two samples were affected by higher doses of radiation than the four other samples.

Gene Ontology analysis of the genes that contribute most to the first principal component shows that " pyruvate metabolism", " glucose metabolism", " carbohydrate metabolism", and " ergosterol biosynthesis" ontologies (here we list only independent ontologies) are over-represented (with p-values less than 10^-10^). The second component is associated with " trehalose biosynthesis", and " carboxylic acid metabolism" ontologies and the third principal component is associated with the KEGG glycolysis pathway. The first three principal components collect 25%, 17% and 11% of the total dispersion.

**Figure 3:**
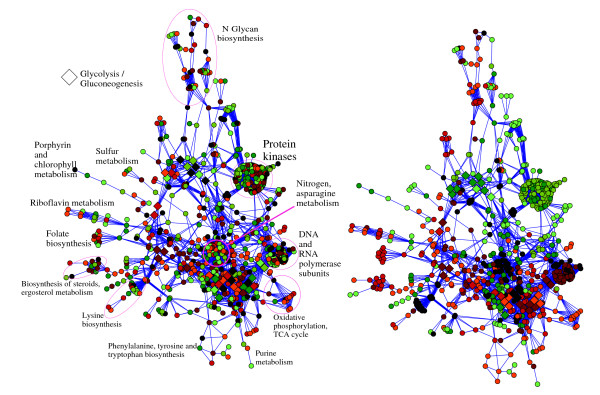
**Representation of the classifiers**. Global connection map of KEGG with mapped coefficients of the decision function obtained by applying a customary linear SVM (**left**) and using high-frequency eigenvalue attenuation (80% of high-frequency eigenvalues have been removed) (**right**). Spectral filtering divided the whole network into modules having coordinated responses, with the activation of low-frequency eigen modes being determined by microarray data. Positive coefficients are marked in red, negative coefficients are in green, and the intensity of the colour reflects the absolute values of the coefficients. Rhombuses highlight proteins participating in the Glycolysis/Gluconeogenesis KEGG pathway. Some other parts of the network are annotated including big highly connected clusters corresponding to proteinkinases and DNA and RNA polymerase sub-units.

The transformation (3) resulting from a step-like attenuation of eigenvalues *φ*_thres _removing 80% of the largest eigenvalues significantly changes the global layout of data (Figure 2, right) but generally preserves the local neighbourhood relationships. The first three principal components collect 28%, 20% and 12% of the total dispersion, which is only slightly higher than the PCA plot of the initial profiles. The general tendency is that the non-irradiated normal samples are more closely grouped, which explains the lower intraclass distance values shown in Figure 1. The principal components in this case allows them to be associated with gene ontologies with higher confidence (for the first component, the p-values are less than 10^-25^). This is a direct consequence of the fact that the principal components are constrained to belong to a subspace of smooth functions on KEGG, giving coherence in terms of pathways to the genes contributing to the components. The first component give "DNA-directed RNA polymerase activity", "RNA polymerase complex" and " protein kinase activity". Figure 3 shows that these are the most connected clusters of the whole KEGG network. The second component is associated with "purine ribonucleotide metabolism", "RNA polymerase complex", "carboxylic acid metabolism" and "acetyl-CoA metabolism" ontologies and also with "Glycolysis/Gluconeogenesis", "Citrate cycle (TCA cycle)" and "Reductive carboxylate cycle (CO2 fixation)" KEGG pathways. The third component is associated with "prenyltransferase activity", "lyase activity" and "aspartate family amino acid metabolism" ontologies and with "N-Glycan biosynthesis", "Glycerophospholipid metabolism", "Alanine and aspartate metabolism" and "riboflavin metabolism" KEGG pathways. Thus, the PCA components of the transformed expression profiles are affected both by network features and by the microarray data.

### Supervised classification

We tested the performance of supervised classification after modifying the distances with a support vector machine (SVM) trained to discriminate irradiated samples from non-irradiated samples. For each change of metric, we estimated the performance of the SVM from the total number of misclassifications and the total hinge loss using a "leave-one-out" (LOO) approach. This approach removes each sample in turn, trains a classifier on the remaining samples and then tests the resulting classifier on the removed sample. For each fold, the regularisation parameter was selected from the training set only by minimising the classification error estimated with an internal LOO experiment. The calculations were carried out using the svmpath package in the R computing environment.

**Figure 4:**
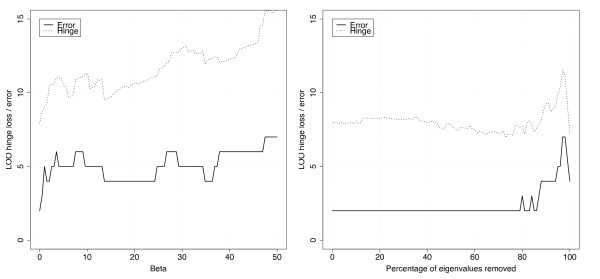
**Supervised classification results**. Performance of the supervised classification when changing the metric with the function *φ*_exp_(*λ*) = exp(-*βλ*) for different values of *β *(left picture), or the function *φ*_thres_(*λ*) = 1(*λ *<*λ*_0_) for different values of *λ*_0 _(i.e., keeping only a fraction of the smallest eigenvalues, right picture). The performance is estimated from the number of misclassifications in a leave-one-out error

Figure 4 shows the classification results for the two high frequency attenuation functions *φ*_exp _and *φ*_thres _with varying parameters. The baseline LOO error is 2 misclassifications for the SVM in the original Euclidean space. For the exponential variant (*φ*_exp_(*λ*) = exp(-*βλ*)), we observe an irregular but certain degradation in performance for positive *β *for both the hinge loss and the misclassification number. This is consistent with the result shown in Figure 1 in which the change of metric towards the first few eigenvectors does not give a geometry coherent with the classification of samples into irradiated and non-irradiated, resulting in a poorer performance in supervised classification as well. For the second variant, in which the expression profiles are projected onto the eigenvector of the graph with the smallest eigenvalues, we observe that the performance remains as accurate as the baseline performance until up to 80% of the eigenvectors are discarded, with the hinge loss even exhibiting a slight minimum in this region. This is consistent with the classes being more clustered in this case than in the original Euclidean space. Overall these results show that classification accuracy can be kept high even when the classifier is constrained to exhibit a certain coherence with the graph structure.

### Interpretation of the SVM classifier

Figure 3 shows the global connection map of KEGG generated from the connection matrix by Cytoscape software [28]. The coefficients of the decision function *v *of equation (9) for the classifier constructed either in the original Euclidean space or after filtering the 80% top spectral components of the expression profiles are shown in colour. We used a color scale from green (negative weights) to red (positive weights) to provide an easy visualisation of the classifier main features. Both classifiers give the same classification error but the classifier constructed using the network structure can be more naturally interpreted, as the classifier variables are grouped according to their participation in the network modules.

Although from a biological point of view, very little can be learned from the classifier obtained in the original Euclidean space (Figure 3, left), it is indeed possible to distinguish several features of interest for the classifier obtained in the second case (Figure 3, right). First, oxidative phosphorylation is found among the pathways with the most positive weights, which is consistent with previous analyses showing that this pathway tends to be up-regulated after irradiation [26]. An important cluster involving the DNA and RNA polymerases is also found to bear weights slightly above average in these experiments. Several studies have previously reported the induction of genes involved in replication and repair after high doses of irradiation [29], but the detection of such an induction at the low irradiation doses used in the present biological experiments is rather interesting. The strongly negative landscape of weights in the protein kinases cluster has not been seen before and may lead to a new area of biological study. Most of the kinases are involved in signalling pathways, and therefore their low expression levels may have important biological consequences.

**Figure 5:**
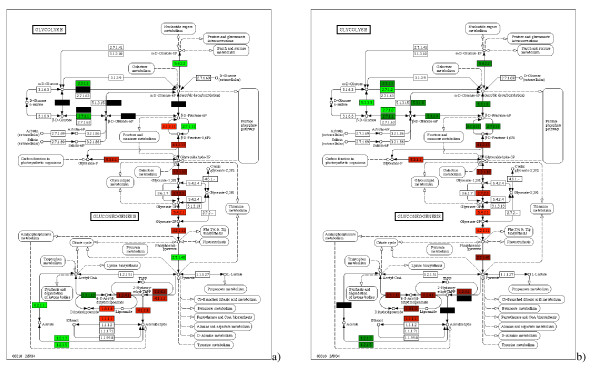
**Glycolysis/gluconeogenesis pathways**. The glycolysis/gluconeogenesis pathways of KEGG with mapped coefficients of the decision function obtained by applying a customary linear SVM (**a**) and using high-frequency eigenvalue attenuation (**b**). The pathways are mutually exclusive in a cell, as clearly highlighted by our algorithm

Figure 3 shows a highlighted pathway named " Glycolysis/Gluconeogenesis" in KEGG. A more detailed view of this pathway is shown in Figure 5. This pathway contains enzymes that are also used in many other KEGG pathways and is therefore situated in the middle and most entangled part of the global network. As already mentioned, this pathway is associated with the first and the third principal components of the initial dataset. The pathway actually contains two alternative sub-pathways that are affected differentially. Over-expression in the gluconeogenesis pathway seems to be characteristic of irradiated samples, whereas glycolysis has a low level of expression in that case. This shift can be observed by changing from anaerobic to aerobic growth conditions (called diauxic shift). The reconstruction of this from our data with no prior input of this knowledge strongly confirms the relevance of our analysis method. It also shows that analysing expression in terms of the global up- or down-regulation of entire pathways as defined, for example, by KEGG, could be misleading as there are many antagonist processes that take place within pathways. By representing KEGG as a large network instead of a set of pathways, our approach helps maintaining the biochemical relationships between genes out of the constraints of pathway limits. Once a classifier has been built using a priori the knowledge of the network, the interpretation of the results (which genes contribute the most to the classification) can be performed through visualisation of known biochemical pathways, or extraction of gene clusters with similar contribution to the classifier. Importantly these gene clusters result from a combined analysis of the gene network and the gene expression data, and not from a prior analysis of the gene network alone.

**Figure 6:**
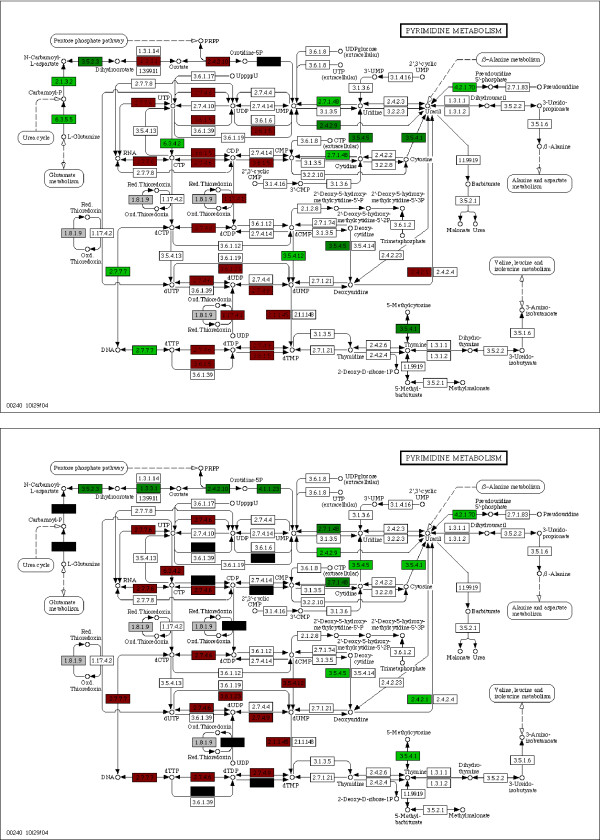
**Pyrimidine metabolism pathways**. The Pyrimidine Metabolism pathways of the separator obtained with an Euclidean linear SVM (**up**) and our modified algorithm (**down**).

Figure 6 shows the weights of the two classifiers on the genes involved in pyrimidine metabolism which is another pathway of interest.

## Discussion

Our algorithm constructs a classifier in which the predictor variables are grouped according to their neighborhood relations in the network. We assume that the genes close on the network are likely to contribute similarly to the prediction function. Our working hypothesis is that *the genes close on the network should have similar expression profiles*. This hypothesis was validated in several studies that demonstrate that co-expressed genes tend to have similar biological functions and vice versa (e.g., [30]). Our mathematical framework based on spectral decomposition helps to systematically exploit this experimental fact and include it into data analysis.

Nevertheless, one must understand that this tendency is only a trend, valid when we take the average on a large scale. It is of course possible to find many local exceptions to this trend, for example when a signaling pathway or a metabolic cascade is influenced by over- or under-expression of only one regulator without systematically affecting the expression of the rest of the pathway participants. Thus, our technique is rather coarse-grained, it does not allow to infer a precise network logic but rather detects *average *excitation of relatively big network modules.

In our example we use a metabolic network as gene network. Our hypothesis here is based on the fact that for a smooth synthesis flow, all enzymes required for a metabolic cascade should be present in sufficient quantities, i.e., stably expressed. On the opposite, various sensor and feedback mechanisms ensure that for inactive metabolic cascades the expression of corresponding enzymes remains low. If this is true *on average *then our technique will help to highlight active and inactive parts of the network. Several previous studies have highlighted the significant correlation that exists between gene expression and distance over the metabolic network, thus justifying our attempt [15,17,23-25]. For other network types, like transcriptional regulatory or signalling network, more elaborated measures of " smoothness" are certainly needed to take into account signs and directions of individual gene interactions.

Our working hypothesis motivates the filtering of gene expression profile in order to remove the noisy high-frequency modes of the network. Therefore, the variation of the weights of the classifier along the graph are of low frequency and should allow grouping of variables, which is a very useful feature of the resulting classification function as the function becomes meaningful for interpreting and suggesting biological factors that cause the class separation. It allows classifications based on functions, pathways and network modules rather than on individual genes. Classification based on pathways and network modules should lead to a more robust behaviour of the classifier in independent tests with equal if not better classification results. Our results on the dataset we analysed show only a slight improvement, although this may be due to its limited size. The two samples with different experimental settings are systematically misclassified in both the initial and our smoothed classifier which means that they probably are members of a " third" class which should be treated differently. Introduction of network topology can not resolve this issue but can help to understand which part of the network differentiate the outliers from the other members of the same class.

Interestingly, the constraint we impose on the smoothness of the classifier can also be justified mathematically in the context of *regularisation *for statistical estimation. Classification of microarray data is an extremely challenging problem because it usually involves a small number of samples in large dimension. Most statistical procedures developed in this context involve some form of complexity reduction by imposing some constraints on the classifier. For example, perhaps the most widely-used complexity reduction method for microarray data is to impose that the classifier has only a small number of non-zero weights, which in practice amounts to selecting a small number of genes. Mathematically speaking, this means constraining the *L*_0 _norm of the classifier to be small (the *L*_0 _norm of a vector being the number of non-zero components). Alternatively, methods like SVM constrain the *L*_2 _norm of the classifier to be small. Our method can therefore be seen as just constraining a different norm of the classifier, for the purpose of regularisation. Of course the choice of regularisation should be related to the problem at hand: it corresponds to our prior belief of what the optimal classifier, that would be discovered if enough samples were available, looks like. Performing feature selection implicitly corresponds to the assumption that the optimal classifier relies on a small number of genes, which can be a reasonable assumption in some cases. Our focus on the smoothness of the classifier on the gene network corresponds to a different implicit assumption, namely, that the optimal classifier is likely to be so. This is justified in many cases because the classes of samples to be predicted generally correspond to differences in the regulation of one or several pathways. Of course if this turns out not to be the case, reducing the effect of regularisation by decreasing the parameter *C *in (9) allows a non-smooth classifier to be learned as well.

An important remark to bear in mind when interpreting pictures such as Figures 3 and 5 is that the colors represent the weights of the classifier, and not gene expression levels. There is of course a relationship between the classifier weights and the typical expression levels of genes in irradiated and non-irradiated samples: irradiated samples tend to have expression profiles positively correlated with the classifier, while non-irradiated samples tend to be negatively correlated. Roughly speaking, the classifier tries to find a smooth function that has this property. This means in particular that the pictures provide virtually no information regarding the over- or under-expression of *individual genes*, which is the cost to pay to obtain instead an interpretation in terms of more *global pathways*. Constraining the classifier to rely on just a few genes would have a similar effect of reducing the complexity of the problem, but would lead to a more difficult interpretation in terms of pathways.

An important advantage of our approach over other pathway-based clustering methods is that we consider the network modules that naturally appear from spectral analysis rather than a historically defined separation of the network into pathways. Thus, pathways cross-talking is taken into account, which is difficult to do using other approaches. It can however be noticed that the implicit decomposition into pathways that we obtain is biased by the very incomplete knowledge of the network and that certain regions of the network are better understood, leading to a higher connection concentration.

Another important feature of this approach is that we make no strong assumption on the nature of the graph, and that the method can in principle be applied with a variety of other graphs, such as protein-protein interaction networks or co-expression networks. We leave this avenue open for future research.

On the other hand, like most approaches aiming at comparing expression data with gene networks such as KEGG, the scope of this work is limited by two important constraints. First the gene network we use is only a convenient but rough approximation to describe complex biochemical processes; second, the transcriptional analysis of a sample can not give any information regarding post-transcriptional regulation and modifications. Nevertheless, we believe that our basic assumptions remain valid, in that we assume that the expression of the genes belonging to the same metabolic pathways module are coordinately regulated. Our interpretation of the results supports this assumption.

Another important caveat is that we simplify the network description as an undirected graph of interactions. Although this would seem to be relevant for simplifying the description of, e.g., protein-protein interaction networks, in reality metabolic networks have a more complex nature. Similarly, gene regulation networks are influenced by the direction, sign and importance of the interaction. Although the incorporation of weights into the Laplacian (equation 1) is straightforward and allows the extension of the approach to weighted undirected graphs, the choice of the weights remains delicate since the importance of an interaction may be difficult to quantify. Conversely the directions and signs that accompany signalling or regulatory pathways are generally known, but their incorporation requires more work. It could nevertheless lead to important advances for the interpretation of microarray data in cancer studies, for example.

## Conclusion

We have presented a general framework to analyse gene expression data when a gene network is known *a priori*. The approach involves the attenuation of the high-frequency content of the gene expression vectors with respect to the graph. We derived algorithms for unsupervised clustering and supervised classification, which enforce some level of smoothness on the gene network for the classifier. This enforcement can be considered as a means of reducing the high dimension of the variable space, using the available knowledge about gene network. No prior decomposition of the gene network into modules or pathways is needed, and the method can work in principle with a variety of gene networks.

## Methods

In this section, we explain how a gene expression vector can be decomposed with respect to the eigenfunctions of a gene network, and how to derive unsupervised and supervised classification algorithms from this decomposition. Before describing the technical details of the method, we start by a brief non-technical overview of the approach.

### Overview of the method

**Figure 7:**
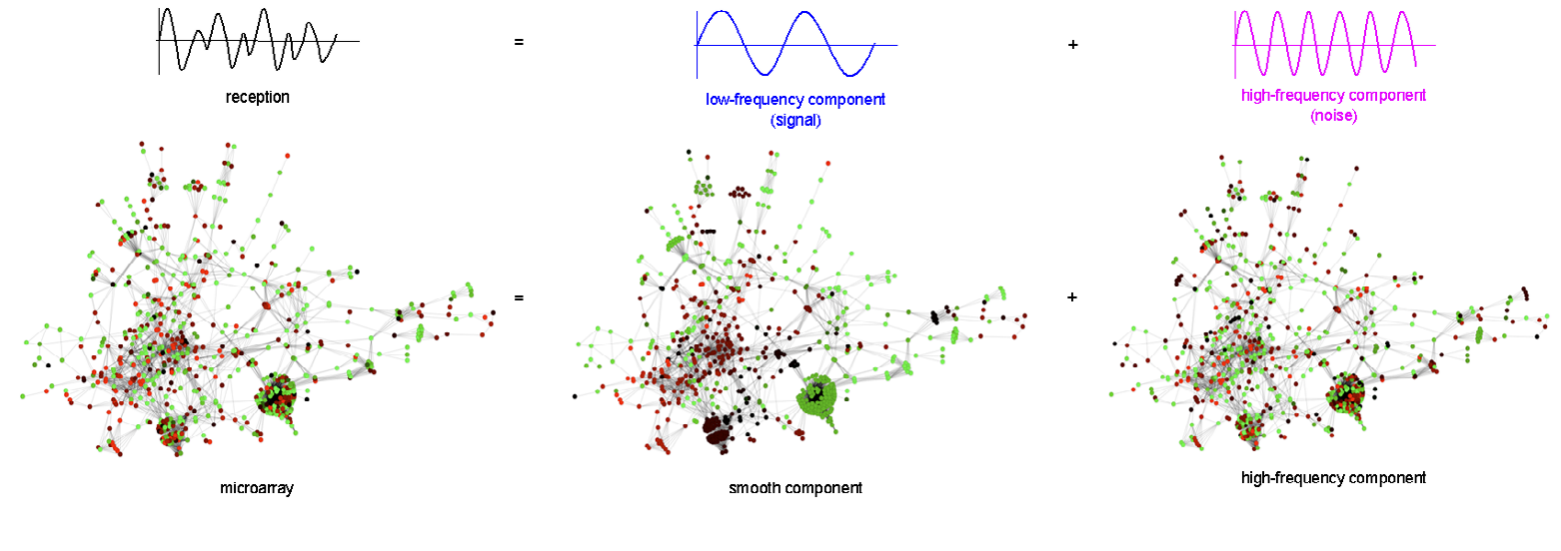
**Decomposition of a gene expression profile**. Following the idea of Fourier decomposition (above), we can decompose a gene expression profile, here the first non-irradiated microarray sample from our data set, into two parts: the smooth component and the high-frequency component. We can then apply some filtering to attenuate or cancel the effect of the high-frequency component.

In this section we briefly outline the main features of our approach. We propose a general mathematical formalism to include *a priori *the knowledge of a gene network for the analysis of gene expression data. The method is independent of the nature of the network, although we focus on the gene metabolic network as an illustration in this paper. It is based on the hypothesis that genes close on the network are likely to be co-expressed, and consequently that a biologically relevant signal can be extracted from noisy gene expression measurement by removing the "high-frequency" components of the gene expression vector over the gene network. The extraction of the low-frequency component of a vector is a classical operation in signal processing (see, e.g., Figure 7), that can be adapted to our problem using discrete Fourier transforms and spectral graph analysis.

We show how this idea can be adapted to solve the problem of supervised classification of samples based on their gene expression microarray profiles. This is achieved optimising a linear classifier such that the weights of genes linked together in the network tend to be similar, i.e., by forcing nearby genes to have similar contribution to the decision function. The resulting classifier can thereafter be easily interpreted by visual inspection of the weights over the gene network, or subsequent extraction of clusters of genes on the network with similar contributions.

### Spectral decomposition of gene expression profiles

We consider a finite set of genes *V *of cardinality |*V*| = *n*. The available gene network is represented by an undirected graph *G *= (*V*, *E*) without loop and multiple edges, in which the set of vertices *V *is the set of genes and *E *⊂ *V *× *V *is the list of edges. We will use the notation *u *~ *v *to indicate that two genes *u *and *v *are neighbors in the graph, that is, (*u*, *v*) ∈ *E*. For any gene *u*, we denote the degree of *u *in the graph by *d*_*u*_, that is, its neighbour number. Gene expression profiling gives a value of expression *f*(*u*) for each gene *u*, and is therefore represented by a function *f*: *V *→ ℝ.

The Laplacian of the graph *G *is the *n *× *n *matrix [31]:

∀u,v∈V,L(u,v)={duif u=v,−1if u~v,0otherwise.      (1)
 MathType@MTEF@5@5@+=feaafiart1ev1aaatCvAUfKttLearuWrP9MDH5MBPbIqV92AaeXatLxBI9gBaebbnrfifHhDYfgasaacH8akY=wiFfYdH8Gipec8Eeeu0xXdbba9frFj0=OqFfea0dXdd9vqai=hGuQ8kuc9pgc9s8qqaq=dirpe0xb9q8qiLsFr0=vr0=vr0dc8meaabaqaciaacaGaaeqabaqabeGadaaakeaafaqabeqacaaabaGaeyiaIiIaemyDauNaeiilaWIaemODayNaeyicI4SaemOvayLaeiilaWcabaGaemitaWKaeiikaGIaemyDauNaeiilaWIaemODayNaeiykaKIaeyypa0ZaaiqabeaafaqaaeWacaaabaGaemizaq2aaSbaaSqaaiabdwha1bqabaaakeaacqqGPbqAcqqGMbGzcqqGGaaicqWG1bqDcqGH9aqpcqWG2bGDcqGGSaalaeaacqGHsislcqaIXaqmaeaacqqGPbqAcqqGMbGzcqqGGaaicqWG1bqDcqGG+bGFcqWG2bGDcqGGSaalaeaacqaIWaamaeaacqqGVbWBcqqG0baDcqqGObaAcqqGLbqzcqqGYbGCcqqG3bWDcqqGPbqAcqqGZbWCcqqGLbqzcqGGUaGlaaaacaGL7baaaaGaeeiiaaIaaCzcaiaaxMaadaqadaqaaiabigdaXaGaayjkaiaawMcaaaaa@6658@

**Figure 8:**
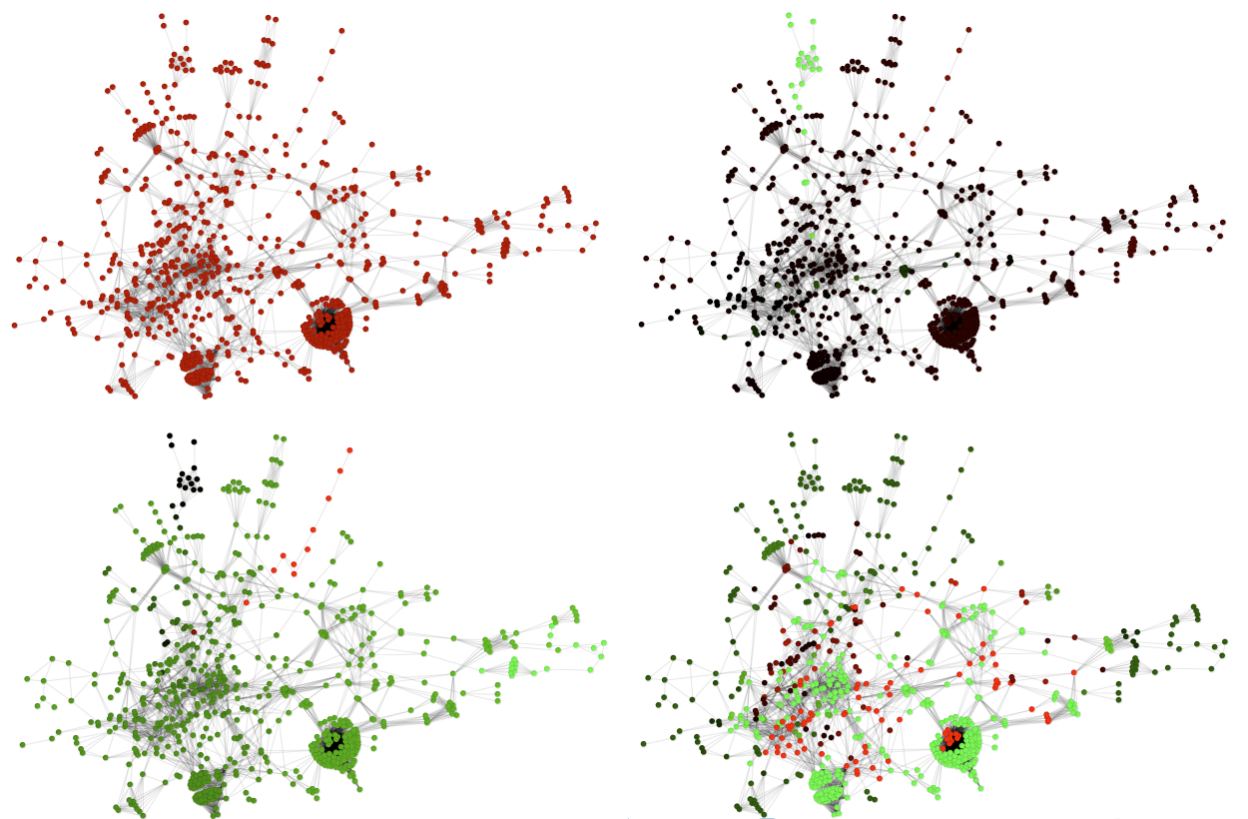
**Example of Laplacian eigenvectors**. Here are four example of Laplacian eigenvectors of the main component of KEGG. The colours correspond to the coefficients of the eigenvectors: positive coefficients are marked in red, negative coefficients are in green, and the intensity of the colour reflects the absolute values of the coefficients. On the upper-left side is the eigenvector associated with the smallest eigenvalue, on the upper-right side the one associated with the second smallest eigenvalues, on the lower-left side, the one associated with the third smallest eigenvalue while on the lower-right side is the one associated with the largest eigenvalue. The larger the eigenvalue, the less smooth the corresponding eigenvector.

The Laplacian is a central concept in spectral graph theory [32] and shares many properties with the Laplace operator on Riemannian manifolds. *L *is known to be symmetric positive semidefinite and singular. We denote its eigenvalues by 0 = *λ*_1 _≤ ... ≤ *λ*_*n *_and the corresponding eigenvectors by *e*_1_,...,*e*_*n*_. The multiplicity of 0 as an eigenvalue is equal to the number of connected components of the graph, and the corresponding eigenvectors are constant on each connected component. The eigen-basis of *L *forms a Fourier basis and a natural theory for Fourier analysis and spectral decomposition on graphs can thus be derived [31]. Essentially, the eigenvectors with increasing eigenvalues tend to vary more abruptly on the graph, and the smoothest functions (constant on each connected component) are associated with the smallest (zero) eigenvalue. For a good example, see Figure 8. In particular, the Fourier transform f^
 MathType@MTEF@5@5@+=feaafiart1ev1aaatCvAUfKttLearuWrP9MDH5MBPbIqV92AaeXatLxBI9gBaebbnrfifHhDYfgasaacH8akY=wiFfYdH8Gipec8Eeeu0xXdbba9frFj0=OqFfea0dXdd9vqai=hGuQ8kuc9pgc9s8qqaq=dirpe0xb9q8qiLsFr0=vr0=vr0dc8meaabaqaciaacaGaaeqabaqabeGadaaakeaacuWGMbGzgaqcaaaa@2E11@ ∈ ℝ^*n *^of any expression profile *f *is defined by:

f^i=∑u∈Vei(u)f(u),i=1,...,n.
 MathType@MTEF@5@5@+=feaafiart1ev1aaatCvAUfKttLearuWrP9MDH5MBPbIqV92AaeXatLxBI9gBaebbnrfifHhDYfgasaacH8akY=wiFfYdH8Gipec8Eeeu0xXdbba9frFj0=OqFfea0dXdd9vqai=hGuQ8kuc9pgc9s8qqaq=dirpe0xb9q8qiLsFr0=vr0=vr0dc8meaabaqaciaacaGaaeqabaqabeGadaaakeaafaqabeqacaaabaGafmOzayMbaKaadaWgaaWcbaGaemyAaKgabeaakiabg2da9maaqafabaGaemyzau2aaSbaaSqaaiabdMgaPbqabaGccqGGOaakcqWG1bqDcqGGPaqkcqWGMbGzcqGGOaakcqWG1bqDcqGGPaqkcqGGSaalaSqaaiabdwha1jabgIGiolabdAfawbqab0GaeyyeIuoaaOqaaiabdMgaPjabg2da9iabigdaXiabcYcaSiabc6caUiabc6caUiabc6caUiabcYcaSiabd6gaUbaacqGGUaGlaaa@4C77@

The eigenvectors of *L *form an orthonormal basis and the expression profile *f *can therefore be recovered from its Fourier transform f^
 MathType@MTEF@5@5@+=feaafiart1ev1aaatCvAUfKttLearuWrP9MDH5MBPbIqV92AaeXatLxBI9gBaebbnrfifHhDYfgasaacH8akY=wiFfYdH8Gipec8Eeeu0xXdbba9frFj0=OqFfea0dXdd9vqai=hGuQ8kuc9pgc9s8qqaq=dirpe0xb9q8qiLsFr0=vr0=vr0dc8meaabaqaciaacaGaaeqabaqabeGadaaakeaacuWGMbGzgaqcaaaa@2E11@ by the simple formula:

f=∑i=1nf^iei.     (2)
 MathType@MTEF@5@5@+=feaafiart1ev1aaatCvAUfKttLearuWrP9MDH5MBPbIqV92AaeXatLxBI9gBaebbnrfifHhDYfgasaacH8akY=wiFfYdH8Gipec8Eeeu0xXdbba9frFj0=OqFfea0dXdd9vqai=hGuQ8kuc9pgc9s8qqaq=dirpe0xb9q8qiLsFr0=vr0=vr0dc8meaabaqaciaacaGaaeqabaqabeGadaaakeaacqWGMbGzcqGH9aqpdaaeWbqaaiqbdAgaMzaajaWaaSbaaSqaaiabdMgaPbqabaGccqWGLbqzdaWgaaWcbaGaemyAaKgabeaaaeaacqWGPbqAcqGH9aqpcqaIXaqmaeaacqWGUbGBa0GaeyyeIuoakiabc6caUiaaxMaacaWLjaWaaeWaaeaacqaIYaGmaiaawIcacaGLPaaaaaa@4071@

Like its continuous counterpart, the discrete Fourier transform can be used for smoothing or for extracting features. Here, our hypothesis is that analysing a gene expression profile from its Fourier transform with respect to an *a priori *given gene network is a practical way to decompose the expression profile into biologically interpretable information and filter out the noise. In the next two sections we illustrate the potential applications of this approach by describing how this leads to a natural definition for distances between expression profiles, and how this distance can be used for classification or regression purposes.

### Deriving a metric for expression profiles

The definition of new metrics on expression profiles that incorporate information encoded in the graph structure is a first possible application of the spectral decomposition. Following the classical methodology in Fourier analysis, we assume that the signal captured in the low-frequency component of the expression profiles contains the most biologically relevant information, particularly the general expression trends, whereas the high-frequency components are more likely measurement noise. For example, the low-frequency component of an expression vector on the gene metabolic network should reveal areas of positive and negative expression on the graph that are likely to correspond to the activation or inhibition of specific branches of the graph. We can translate this idea mathematically by considering the following class of transformations for expression profiles:

∀f∈ℝV,Sφ(f)=∑i=1nf^iφ(λi)ei,     (3)
 MathType@MTEF@5@5@+=feaafiart1ev1aaatCvAUfKttLearuWrP9MDH5MBPbIqV92AaeXatLxBI9gBaebbnrfifHhDYfgasaacH8akY=wiFfYdH8Gipec8Eeeu0xXdbba9frFj0=OqFfea0dXdd9vqai=hGuQ8kuc9pgc9s8qqaq=dirpe0xb9q8qiLsFr0=vr0=vr0dc8meaabaqaciaacaGaaeqabaqabeGadaaakeaafaqabeqacaaabaGaeyiaIiIaemOzayMaeyicI48efv3ySLgznfgDOjdaryqr1ngBPrginfgDObcv39gaiqaacqWFDeIudaahaaWcbeqaaiabdAfawbaakiabcYcaSaqaaiabdofatnaaBaaaleaaiiGacqGFgpGzaeqaaOGaeiikaGIaemOzayMaeiykaKIaeyypa0ZaaabCaeaacuWGMbGzgaqcamaaBaaaleaacqWGPbqAaeqaaOGae4NXdyMaeiikaGIae43UdW2aaSbaaSqaaiabdMgaPbqabaGccqGGPaqkcqWGLbqzdaWgaaWcbaGaemyAaKgabeaaaeaacqWGPbqAcqGH9aqpcqaIXaqmaeaacqWGUbGBa0GaeyyeIuoaaaGccqGGSaalcaWLjaGaaCzcamaabmaabaGaeG4mamdacaGLOaGaayzkaaaaaa@5CA2@

where *φ*: ℝ^+ ^→ ℝ is a non-increasing function that quantifies how each frequency is attenuated. For example, if we take *φ*(*λ*) = 1 for all *λ*, we get from (2) that the profile does not change that is, *S*_*φ *_(*f*) = *f*. However, if we take:

φthres(λ)={1if 0≤λ≤λ0,0if λ>λ0,     (4)
 MathType@MTEF@5@5@+=feaafiart1ev1aaatCvAUfKttLearuWrP9MDH5MBPbIqV92AaeXatLxBI9gBaebbnrfifHhDYfgasaacH8akY=wiFfYdH8Gipec8Eeeu0xXdbba9frFj0=OqFfea0dXdd9vqai=hGuQ8kuc9pgc9s8qqaq=dirpe0xb9q8qiLsFr0=vr0=vr0dc8meaabaqaciaacaGaaeqabaqabeGadaaakeaaiiGacqWFgpGzdaWgaaWcbaGaeeiDaqNaeeiAaGMaeeOCaiNaeeyzauMaee4CamhabeaakiabcIcaOiab=T7aSjabcMcaPiabg2da9maaceqabaqbaeaabiGaaaqaaiabigdaXaqaaiabbMgaPjabbAgaMjabbccaGiabicdaWiabgsMiJkab=T7aSjabgsMiJkab=T7aSnaaBaaaleaacqaIWaamaeqaaOGaeiilaWcabaGaeGimaadabaGaeeyAaKMaeeOzayMaeeiiaaIae83UdWMaeyOpa4Jae83UdW2aaSbaaSqaaiabicdaWaqabaGccqGGSaalaaaacaGL7baacaWLjaGaaCzcamaabmaabaGaeGinaqdacaGLOaGaayzkaaaaaa@57CD@

we produce a low-pass filter that removes all the frequencies from *f *above the threshold *λ*_0_. Finally, a function of the form:

*φ*_exp_(*λ*) = exp(-*βλ*),     (5)

for some *β *> 0, keeps all the frequencies but strongly attenuates the high-frequency components. If *S*_*φ *_(*f*) includes the biologically relevant part of the expression profile, we can compare two expression profiles *f *and *g *through their representations *S*_*φ *_(*f*) and *S*_*φ *_(*g*). This leads to the following metric between the profiles:

dφ(f,g)2=‖Sφ(f)−Sφ(g)‖2=∑i=1n(f^i−g^i)2φ(λi)2.
 MathType@MTEF@5@5@+=feaafiart1ev1aaatCvAUfKttLearuWrP9MDH5MBPbIqV92AaeXatLxBI9gBaebbnrfifHhDYfgasaacH8akY=wiFfYdH8Gipec8Eeeu0xXdbba9frFj0=OqFfea0dXdd9vqai=hGuQ8kuc9pgc9s8qqaq=dirpe0xb9q8qiLsFr0=vr0=vr0dc8meaabaqaciaacaGaaeqabaqabeGadaaakeaafaqadeGabaaabaGaemizaq2aaSbaaSqaaGGaciab=z8aMbqabaGccqGGOaakcqWGMbGzcqGGSaalcqWGNbWzcqGGPaqkdaahaaWcbeqaaiabikdaYaaakiabg2da9maafmaabaGaem4uam1aaSbaaSqaaiab=z8aMbqabaGccqGGOaakcqWGMbGzcqGGPaqkcqGHsislcqWGtbWudaWgaaWcbaGae8NXdygabeaakiabcIcaOiabdEgaNjabcMcaPaGaayzcSlaawQa7amaaCaaaleqabaGaeGOmaidaaaGcbaGaeyypa0ZaaabCaeaacqGGOaakcuWGMbGzgaqcamaaBaaaleaacqWGPbqAaeqaaOGaeyOeI0Iafm4zaCMbaKaadaWgaaWcbaGaemyAaKgabeaakiabcMcaPmaaCaaaleqabaGaeGOmaidaaOGae8NXdyMaeiikaGIae83UdW2aaSbaaSqaaiabdMgaPbqabaGccqGGPaqkdaahaaWcbeqaaiabikdaYaaaaeaacqWGPbqAcqGH9aqpcqaIXaqmaeaacqWGUbGBa0GaeyyeIuoakiabc6caUaaaaaa@634A@

We note that this Euclidean metric over expression profiles is associated with the following inner products:

〈f,g〉φ=∑i=1nf^ig^iφ(λi)2=∑i=1nf⊤eiei⊤gφ(λi)2=f⊤Kφg,     (6)
 MathType@MTEF@5@5@+=feaafiart1ev1aaatCvAUfKttLearuWrP9MDH5MBPbIqV92AaeXatLxBI9gBaebbnrfifHhDYfgasaacH8akY=wiFfYdH8Gipec8Eeeu0xXdbba9frFj0=OqFfea0dXdd9vqai=hGuQ8kuc9pgc9s8qqaq=dirpe0xb9q8qiLsFr0=vr0=vr0dc8meaabaqaciaacaGaaeqabaqabeGadaaakeaafaqadeWabaaabaGaeyykJeUaemOzayMaeiilaWIaem4zaCMaeyOkJe=aaSbaaSqaaGGaciab=z8aMbqabaGccqGH9aqpdaaeWbqaaiqbdAgaMzaajaWaaSbaaSqaaiabdMgaPbqabaGccuWGNbWzgaqcamaaBaaaleaacqWGPbqAaeqaaOGae8NXdyMaeiikaGIae83UdW2aaSbaaSqaaiabdMgaPbqabaGccqGGPaqkdaahaaWcbeqaaiabikdaYaaaaeaacqWGPbqAcqGH9aqpcqaIXaqmaeaacqWGUbGBa0GaeyyeIuoaaOqaaiabg2da9maaqahabaGaemOzay2aaWbaaSqabeaat0uy0HwzTfgDPnwyZaqeg0uy0HwzTfgDPnwyZaaceaGae4hPIGkaaOGaemyzau2aaSbaaSqaaiabdMgaPbqabaGccqWGLbqzdaqhaaWcbaGaemyAaKgabaGae4hPIGkaaOGaem4zaCMae8NXdyMaeiikaGIae83UdW2aaSbaaSqaaiabdMgaPbqabaGccqGGPaqkdaahaaWcbeqaaiabikdaYaaaaeaacqWGPbqAcqGH9aqpcqaIXaqmaeaacqWGUbGBa0GaeyyeIuoaaOqaaiabg2da9iabdAgaMnaaCaaaleqabaGae4hPIGkaaOGaem4saS0aaSbaaSqaaiab=z8aMbqabaGccqWGNbWzcqGGSaalaaGaaCzcaiaaxMaadaqadaqaaiabiAda2aGaayjkaiaawMcaaaaa@7EA7@

where Kφ=∑i=1nφ(λi)2eiei⊤
 MathType@MTEF@5@5@+=feaafiart1ev1aaatCvAUfKttLearuWrP9MDH5MBPbIqV92AaeXatLxBI9gBaebbnrfifHhDYfgasaacH8akY=wiFfYdH8Gipec8Eeeu0xXdbba9frFj0=OqFfea0dXdd9vqai=hGuQ8kuc9pgc9s8qqaq=dirpe0xb9q8qiLsFr0=vr0=vr0dc8meaabaqaciaacaGaaeqabaqabeGadaaakeaacqWGlbWsdaWgaaWcbaacciGae8NXdygabeaakiabg2da9maaqadabaGae8NXdyMaeiikaGIae83UdW2aaSbaaSqaaiabdMgaPbqabaGccqGGPaqkdaahaaWcbeqaaiabikdaYaaakiabdwgaLnaaBaaaleaacqWGPbqAaeqaaOGaemyzau2aa0baaSqaaiabdMgaPbqaamrtHrhAL1wy0L2yHndaryqtHrhAL1wy0L2yHndaiqaacqGFKkcQaaaabaGaemyAaKMaeyypa0JaeGymaedabaGaemOBa4ganiabggHiLdaaaa@504C@ is the positive semidefinite matrix obtained by modifying the eigenvalues of *L *through *φ*. For example, taking *φ*(*λ*) = exp(-*βλ*) leads to *K*_*φ *_= exp_*M *_(-*βL*), where exp_*M *_denotes the matrix exponential. This observation shows that working with filtered expression profiles (3) is equivalent to defining a *kernel *(6) over the set of expression profiles, in the context of support vector machines and kernel methods [33,34]. This possibility is further explored in the next section.

### Supervised learning and regression

The construction of predictive models for a property or phenotype of interest from the gene expression profiles of the studied samples is a second possible application of the spectral decomposition of expression profiles on the gene network. Typical applications include predicting cancer diagnosis or prognosis from gene expression data, or discriminating between different treatments applied to micro-organisms. Most approaches presented so far build predictive models from the gene expression alone, and then check whether the predictive model is biologically relevant by studying, for example, whether genes with high weights are located in similar pathways. However the genes often give no clear biological meaning. Here, we propose a method combining both steps in a single predictive model that is trained by forcing some form of biological relevance.

We use linear predictive models to predict a variable of interest *y *from an expression profile *f*. They are obtained by solving the following optimisation problem:

min⁡w∈ℝn∑i=1pl(w⊤fi,yi)+C‖w‖2,     (7)
 MathType@MTEF@5@5@+=feaafiart1ev1aaatCvAUfKttLearuWrP9MDH5MBPbIqV92AaeXatLxBI9gBaebbnrfifHhDYfgasaacH8akY=wiFfYdH8Gipec8Eeeu0xXdbba9frFj0=OqFfea0dXdd9vqai=hGuQ8kuc9pgc9s8qqaq=dirpe0xb9q8qiLsFr0=vr0=vr0dc8meaabaqaciaacaGaaeqabaqabeGadaaakeaadaWfqaqaaiGbc2gaTjabcMgaPjabc6gaUbWcbaGaem4DaCNaeyicI48efv3ySLgznfgDOjdaryqr1ngBPrginfgDObcv39gaiqaacqWFDeIudaahaaadbeqaaiabd6gaUbaaaSqabaGcdaaeWbqaaiabdYgaSjabcIcaOiabdEha3naaCaaaleqabaWenfgDOvwBHrxAJf2maeXbnfgDOvwBHrxAJf2maGqbaiab+rQiOcaakiabdAgaMnaaBaaaleaacqWGPbqAaeqaaOGaeiilaWIaemyEaK3aaSbaaSqaaiabdMgaPbqabaGccqGGPaqkcqGHRaWkcqWGdbWqdaqbdaqaaiabdEha3bGaayzcSlaawQa7amaaCaaaleqabaGaeGOmaidaaaqaaiabdMgaPjabg2da9iabigdaXaqaaiabdchaWbqdcqGHris5aOGaeiilaWIaaCzcaiaaxMaadaqadaqaaiabiEda3aGaayjkaiaawMcaaaaa@6A92@

where (*f*_1_,*y*_1_),...,(*f*_*p*_, *y*_*p*_) is a training set of profiles containing the variable *y *to be predicted, and *l *is a loss function that measures the cost of predicting *w*^⊤ ^*f*_*i *_instead of *y*_*i*_. For example, the popular support vector machine [33-35] is a particular case of equation (7) in which *y *can take values in -1, +1 and *l*(*u*, *y*) = max(0,1 - *yu*) is the hinge loss function; ridge regression is obtained for *y *∈ ℝ by taking *l*(*u*, *y*) = (*u *- *y*)^2 ^[36].

Here, we do not apply algorithms of the form (7) directly to the expression profiles *f*, but to their images *S*_*φ *_(*f*). That is, we consider the problem:

min⁡w∈ℝn∑i=1pl(w⊤Sφ(fi),yi)+C‖w‖2.     (8)
 MathType@MTEF@5@5@+=feaafiart1ev1aaatCvAUfKttLearuWrP9MDH5MBPbIqV92AaeXatLxBI9gBaebbnrfifHhDYfgasaacH8akY=wiFfYdH8Gipec8Eeeu0xXdbba9frFj0=OqFfea0dXdd9vqai=hGuQ8kuc9pgc9s8qqaq=dirpe0xb9q8qiLsFr0=vr0=vr0dc8meaabaqaciaacaGaaeqabaqabeGadaaakeaadaWfqaqaaiGbc2gaTjabcMgaPjabc6gaUbWcbaGaem4DaCNaeyicI48efv3ySLgznfgDOjdaryqr1ngBPrginfgDObcv39gaiqaacqWFDeIudaahaaadbeqaaiabd6gaUbaaaSqabaGcdaaeWbqaaiabdYgaSjabcIcaOiabdEha3naaCaaaleqabaWenfgDOvwBHrxAJf2maeXbnfgDOvwBHrxAJf2maGqbaiab+rQiOcaakiabdofatnaaBaaaleaaiiGacqqFgpGzaeqaaOGaeiikaGIaemOzay2aaSbaaSqaaiabdMgaPbqabaGccqGGPaqkcqGGSaalcqWG5bqEdaWgaaWcbaGaemyAaKgabeaakiabcMcaPiabgUcaRiabdoeadnaafmaabaGaem4DaChacaGLjWUaayPcSdWaaWbaaSqabeaacqaIYaGmaaaabaGaemyAaKMaeyypa0JaeGymaedabaGaemiCaahaniabggHiLdGccqGGUaGlcaWLjaGaaCzcamaabmaabaGaeGioaGdacaGLOaGaayzkaaaaaa@6F6D@

We claim that by solving (8) we will find a linear predictor over the original expression profiles that tend to be smooth on the gene network. Indeed, for any *w *∈ ℝ^*d*^, let v=Kφ1/2w
 MathType@MTEF@5@5@+=feaafiart1ev1aaatCvAUfKttLearuWrP9MDH5MBPbIqV92AaeXatLxBI9gBaebbnrfifHhDYfgasaacH8akY=wiFfYdH8Gipec8Eeeu0xXdbba9frFj0=OqFfea0dXdd9vqai=hGuQ8kuc9pgc9s8qqaq=dirpe0xb9q8qiLsFr0=vr0=vr0dc8meaabaqaciaacaGaaeqabaqabeGadaaakeGabaaMniabdAha2jabg2da9iabdUealnaaDaaaleaaiiGacqWFgpGzaeaacqaIXaqmcqGGVaWlcqaIYaGmaaGccqWG3bWDaaa@3724@. We first observe that for any *f *∈ ℝ^*n*^:

w⊤Sφ(f)=w⊤∑i=1nf^iφ(λi)ei=f⊤∑i=1neiφ(λi)ei⊤w=f⊤Kφ1/2w=f⊤v,
 MathType@MTEF@5@5@+=feaafiart1ev1aaatCvAUfKttLearuWrP9MDH5MBPbIqV92AaeXatLxBI9gBaebbnrfifHhDYfgasaacH8akY=wiFfYdH8Gipec8Eeeu0xXdbba9frFj0=OqFfea0dXdd9vqai=hGuQ8kuc9pgc9s8qqaq=dirpe0xb9q8qiLsFr0=vr0=vr0dc8meaabaqaciaacaGaaeqabaqabeGadaaakeaafaqadeabbaaaaeaacqWG3bWDdaahaaWcbeqaamrtHrhAL1wy0L2yHndaryqtHrhAL1wy0L2yHndaiqaacqWFKkcQaaGccqWGtbWudaWgaaWcbaacciGae4NXdygabeaakiabcIcaOiabdAgaMjabcMcaPiabg2da9iabdEha3naaCaaaleqabaGae8hPIGkaaOWaaabCaeaacuWGMbGzgaqcamaaBaaaleaacqWGPbqAaeqaaOGae4NXdyMaeiikaGIae43UdW2aaSbaaSqaaiabdMgaPbqabaGccqGGPaqkcqWGLbqzdaWgaaWcbaGaemyAaKgabeaaaeaacqWGPbqAcqGH9aqpcqaIXaqmaeaacqWGUbGBa0GaeyyeIuoaaOqaaiabg2da9iabdAgaMnaaCaaaleqabaGae8hPIGkaaOWaaabCaeaacqWGLbqzdaWgaaWcbaGaemyAaKgabeaakiab+z8aMjabcIcaOiab+T7aSnaaBaaaleaacqWGPbqAaeqaaOGaeiykaKIaemyzau2aa0baaSqaaiabdMgaPbqaaiab=rQiOcaakiabdEha3bWcbaGaemyAaKMaeyypa0JaeGymaedabaGaemOBa4ganiabggHiLdaakeaacqGH9aqpcqWGMbGzdaahaaWcbeqaaiab=rQiOcaakiabdUealnaaDaaaleaacqGFgpGzaeaacqaIXaqmcqGGVaWlcqaIYaGmaaGccqWG3bWDaeaacqGH9aqpcqWGMbGzdaahaaWcbeqaaiab=rQiOcaakiabdAha2jabcYcaSaaaaaa@85FA@

showing that the final predictor obtained by minimizing (8) is equal to *v*^⊤ ^*f*. Second, we note that:

‖w‖2=w⊤w=v⊤Kφ−1v=∑i=1nv^i2φ(λi)2,
 MathType@MTEF@5@5@+=feaafiart1ev1aaatCvAUfKttLearuWrP9MDH5MBPbIqV92AaeXatLxBI9gBaebbnrfifHhDYfgasaacH8akY=wiFfYdH8Gipec8Eeeu0xXdbba9frFj0=OqFfea0dXdd9vqai=hGuQ8kuc9pgc9s8qqaq=dirpe0xb9q8qiLsFr0=vr0=vr0dc8meaabaqaciaacaGaaeqabaqabeGadaaakeaafaqadeWabaaabaWaauWaaeaacqWG3bWDaiaawMa7caGLkWoadaahaaWcbeqaaiabikdaYaaakiabg2da9iabdEha3naaCaaaleqabaWenfgDOvwBHrxAJf2maeHbnfgDOvwBHrxAJf2maGabaiab=rQiOcaakiabdEha3bqaaiabg2da9iabdAha2naaCaaaleqabaGae8hPIGkaaOGaem4saS0aa0baaSqaaGGaciab+z8aMbqaaiabgkHiTiabigdaXaaakiabdAha2bqaaiabg2da9maaqahabaWaaSaaaeaacuWG2bGDgaqcamaaDaaaleaacqWGPbqAaeaacqaIYaGmaaaakeaacqGFgpGzcqGGOaakcqGF7oaBdaWgaaWcbaGaemyAaKgabeaakiabcMcaPmaaCaaaleqabaGaeGOmaidaaaaaaeaacqWGPbqAcqGH9aqpcqaIXaqmaeaacqWGUbGBa0GaeyyeIuoakiabcYcaSaaaaaa@61BF@

where the last equality remains valid if *K*_*φ *_is not invertible simply by not including in the sum the term *i *for which *φ *(*λ*_*i*_) = 0. This shows that (8) is the equivalent of solving the following problem in the original space:

min⁡v∈ℝn∑i=1pL(v⊤fi,yi)+C∑i:φ(λi)>0v^i2φ(λi)2.     (9)
 MathType@MTEF@5@5@+=feaafiart1ev1aaatCvAUfKttLearuWrP9MDH5MBPbIqV92AaeXatLxBI9gBaebbnrfifHhDYfgasaacH8akY=wiFfYdH8Gipec8Eeeu0xXdbba9frFj0=OqFfea0dXdd9vqai=hGuQ8kuc9pgc9s8qqaq=dirpe0xb9q8qiLsFr0=vr0=vr0dc8meaabaqaciaacaGaaeqabaqabeGadaaakeaadaWfqaqaaiGbc2gaTjabcMgaPjabc6gaUbWcbaGaemODayNaeyicI48efv3ySLgznfgDOjdaryqr1ngBPrginfgDObcv39gaiqaacqWFDeIudaahaaadbeqaaiabd6gaUbaaaSqabaGcdaaeWbqaaiabdYeamjabcIcaOiabdAha2naaCaaaleqabaWenfgDOvwBHrxAJf2maeXbnfgDOvwBHrxAJf2maGqbaiab+rQiOcaakiabdAgaMnaaBaaaleaacqWGPbqAaeqaaOGaeiilaWIaemyEaK3aaSbaaSqaaiabdMgaPbqabaGccqGGPaqkcqGHRaWkcqWGdbWqaSqaaiabdMgaPjabg2da9iabigdaXaqaaiabdchaWbqdcqGHris5aOWaaabuaeaadaWcaaqaaiqbdAha2zaajaWaa0baaSqaaiabdMgaPbqaaiabikdaYaaaaOqaaGGaciab9z8aMjabcIcaOiab9T7aSnaaBaaaleaacqWGPbqAaeqaaOGaeiykaKYaaWbaaSqabeaacqaIYaGmaaaaaaqaaiabdMgaPjabcQda6iab9z8aMjabcIcaOiab9T7aSnaaBaaameaacqWGPbqAaeqaaSGaeiykaKIaeyOpa4JaeGimaadabeqdcqGHris5aOGaeiOla4IaaCzcaiaaxMaadaqadaqaaiabiMda5aGaayjkaiaawMcaaaaa@7D9D@

Thus, the resulting algorithm amounts to finding a linear predictor *v *that minimises the loss function of interest *l *regularised by a term that penalises the high-frequency components of *v*. This is different from the classical regularisation ||*v*||^2 ^used in (7) that only focuses on the norm of *v*. As a result, the linear predictor *v *can be made smoother on the gene network by increasing the parameter *C*. This allows the prior knowledge to be direcly included because genes in similar pathways would be expected to contribute similarly to the predictive model.

There are two consequences of this procedure. Firstly, if the true predictor really is smooth on the graph, the formulation (9) can help the algorithm focus on plausible models even with very little training data, resulting in a better estimation. As a result, we can expect a better predictive performance. Secondly, by forcing the predictive model *v *to be smooth on the graph, biological interpretation of the model should be easier by inspecting the areas of the graph in which the predictor is strongly positive or negative. Thus the model should be easier to interpret than models resulting from the direct optimisation of equation (7).

## Authors' contributions

FR, with the help and under the supervision of JPV and EB, developed the method and implemented the unsupervised and supervised classification algorithms. AZ participated in the conception of the method, performed the PGA analysis with its interpretation and provided the classifier Cytoscape image. MD provided the data set and did the biological interpretation of the supervised classification results. All authors contributed to writing the text, read and approved the final manuscript.
